# Blue-light-activated phototropin2 trafficking from the cytoplasm to Golgi/post-Golgi vesicles

**DOI:** 10.1093/jxb/eru172

**Published:** 2014-05-12

**Authors:** Chhavi Aggarwal, Agnieszka Katarzyna Banaś, Anna Kasprowicz-Maluśki, Carolina Borghetti, Justyna Łabuz, Jerzy Dobrucki, Halina Gabryś

**Affiliations:** ^1^Department of Plant Biotechnology, Faculty of Biochemistry, Biophysics and Biotechnology, Jagiellonian University, Gronostajowa 7, 30–387 Krakow, Poland; ^2^Department of Molecular and Cellular Biology, Faculty of Biology, Adam Mickiewicz University, Umultowska 89, 61–614 Poznan, Poland; ^3^Laboratory of Cell Biophysics, Faculty of Biochemistry, Biophysics and Biotechnology, Jagiellonian University, Gronostajowa 7, 30–387 Krakow, Poland

**Keywords:** *Arabidopsis thaliana*, blue light, degradation, Golgi complex, phototropin, trafficking.

## Abstract

Phototropins are plasma membrane-localized UVA/blue light photoreceptors which mediate phototropism, inhibition of primary hypocotyl elongation, leaf positioning, chloroplast movements, and stomatal opening. Blue light irradiation activates the C-terminal serine/threonine kinase domain of phototropin which autophosphorylates the receptor. *Arabidopsis thaliana* encodes two phototropins, phot1 and phot2. In response to blue light, phot1 moves from the plasma membrane into the cytosol and phot2 translocates to the Golgi complex. In this study the molecular mechanism and route of blue-light-induced phot2 trafficking are demonstrated. It is shown that Atphot2 behaves in a similar manner when expressed transiently under 35S or its native promoter. The phot2 kinase domain but not blue-light-mediated autophosphorylation is required for the receptor translocation. Using co-localization and western blotting, the receptor was shown to move from the cytoplasm to the Golgi complex, and then to the post-Golgi structures. The results were confirmed by brefeldin A (an inhibitor of the secretory pathway) which disrupted phot2 trafficking. An association was observed between phot2 and the light chain2 of clathrin via bimolecular fluorescence complementation. The fluorescence was observed at the plasma membrane. The results were confirmed using co-immunoprecipitation. However, tyrphostin23 (an inhibitor of clathrin-mediated endocytosis) and wortmannin (a suppressor of receptor endocytosis) were not able to block phot2 trafficking, indicating no involvement of receptor endocytosis in the formation of phot2 punctuate structures. Protein turnover studies indicated that the receptor was continuously degraded in both darkness and blue light. The degradation of phot2 proceeded via a transport route different from translocation to the Golgi complex.

## Summary

The blue-light-induced trafficking of the UVA/blue light receptor phototropin2 is shown. Evidence is provided for the presence of two pathways, one directing phototropin2 to the Golgi and post-Golgi vesicles, and the other to degradation.

## Introduction

Many cellular processes in plants depend on sensing and responding to light direction, duration, quantity, and quality. Different classes of photoreceptors have been identified that activate the light-dependent responses in an efficient and accurate manner. One of them is phototropin, a UVA/blue light receptor involved in regulating processes that are important for the promotion of plant growth and for the optimization of photosynthetic efficiency (Briggs, 2001).

Phototropin contains two Per-Arnt-Sim domains (LOV1 and LOV2) at the N-terminus and a serine-threonine kinase domain at the C-terminus. In the absence of blue light, LOV domains non-covalently bind flavin mononucleotide (FMN) as a chromophore. Blue light absorption results in the formation of a covalent adduct between the LOV domain and FMN, finally activating the C-terminal kinase domain which autophosphorylates phototropin (Pfeifer *et al.*, 2010). The two phototropins in *Arabidopsis*, phot1 and phot2, regulate various responses including phototropism (hypocotyl bending), stomatal movements, chloroplast redistribution, the inhibition of primary hypocotyl elongation, leaf positioning, and the stability of resistance protein HRT (Briggs and Christie, 2002; Folta *et al.*, 2003; Inoue *et al.*, 2008; Jeong *et al.*, 2010). Studies from phototropin knockout mutants have shown that phot1 and phot2 play overlapping as well as distinct roles in the control of these responses. The signal transduction from phototropins involves secondary messengers, Ca^2+^, and phosphoinositides (Harada and Shimazaki, 2007; Chen *et al.*, 2008; Aggarwal *et al.*, 2013).

Upon continuous irradiation with blue light, the mRNA levels of phot1 decrease and those of phot2 increase (Labuz *et al.*, 2012). The two phototropins lack membrane-spanning domains but they are mainly associated with the plasma membrane region (Sakamoto and Briggs, 2002; Harada *et al.*, 2003; Kong *et al.*, 2006). Recently, a fraction of phot2 and to a lesser extent a fraction of phot1 were also shown to be present on the chloroplast outer membrane (Kong *et al.*, 2013), but the evidence provided is not entirely convincing. Although both proteins share the same cellular location, blue light changes their localization differently. In the presence of blue light, phot1 moves from the plasma membrane to the cytosol (Sakamoto and Briggs, 2002). Blue-light-activated phot1 internalization has been shown to be phosphorylation dependent (Kaiserli *et al.*, 2009). Recently, Roberts and co-workers (2011) demonstrated phot1 ubiquitination in response to blue light. Interestingly, studies performed on *Arabidopsis* seedlings have shown that phot2, unlike phot1, translocates from the plasma membrane and/or cytoplasm and co-localizes with the Golgi apparatus after blue light irradiation (Kong *et al.*, 2006). The C-terminal kinase moiety determines the specific localization pattern of phototropins in blue light (Aihara *et al.*, 2008). In contrast to phot1, the mechanism and significance of blue-light-dependent phot2 translocation are still unknown.

The function of receptors depends significantly on their number on the cell surface, which is determined by the endocytic and secretory pathway. Receptor endocytosis is an essential mechanism regulating cell signalling from the plasma membrane. The multitude of plant receptors undergoing internalization include auxin transporter PIN proteins (Geldner *et al.*, 2001; Paciorek *et al.*, 2005; Dhonukshe *et al.*, 2007), the brassinosteroid signalling receptor BRI1 (Russinova *et al.*, 2004; Geldner *et al.*, 2007), the defence-inducing receptors FLS2 and LeEix2 (Robatzek *et al.*, 2006), and the metal transporters IRT1 and BOR1 (Takano *et al.*, 2005; Barberon *et al.*, 2011). The initial internalization event at the plasma membrane is followed by a series of transfers where the receptor is directed to different endosomal compartments, involving early endosomes, the *trans*-Golgi network, pre-vacuolar compartments (also called late endosomes, destined for protein degradation), and recycling endosomes (for recycling back to the cell membrane) (Irani and Russinova, 2009).

Although there have been extensive studies on the endocytic pathway, relatively little is known about the regulation of receptors in the secretory pathway. Secretory cargo is made in the endoplasmic reticulum (ER), trafficks through the Golgi, and is packaged into vesicles that are transported to and fuse with either the lysosomes/vacuole or the cell membrane (Viotti *et al.*, 2010). The receptor goes to the plasma membrane either from the *trans*-Golgi network or from pre-vacuolar compartments. How the secreted proteins reach the plasma membrane still needs to be elucidated. Work by Jaiswal *et al.* (2009) showed that the known regulators of endocytosis—clathrin, dynamin, and actin—also control the nature and extent of post-Golgi vesicle exocytosis.

Advanced imaging techniques and various fluorescent proteins have allowed the dynamic observation of receptor trafficking in live cells. Using genetic and chemical approaches, the mechanism and trafficking route of phot2 are proposed. Blue light activates the movement of phot2 from the cytoplasm to the Golgi and the *trans*-Golgi network. This movement is dependent on the kinase domain of the receptor but not on receptor autophosphorylation. Pharmacological agents blocking receptor internalization from the cell surface have no inhibitory effect on phot2 trafficking, excluding the involvement of endocytosis in phot2 translocation. Turnover studies of phot2 suggest the presence of two separate pathways. One directs the receptor to the Golgi complex and the other leads to degradation.

## Materials and methods

### Plant materials

*Arabidopsis thaliana phot1* (At3g45780) mutant seeds were obtained from the NASC (Nottingham Arabidopsis Stock Centre, Nottingham, UK). Seeds were sown on half-strength Murashige and Skoog (MS) medium (MP Biomedicals) and grown under *in vitro* conditions in a growth chamber (Sanyo MLR 350H, Japan) at 23 °C, a photosynthetic photon flux density of 70–100 μmol m^–2^ s^–1^, and a photoperiod of 10h light/14h dark. Experiments were performed on fully grown leaves of 5- to 6-week-old *Arabidopsis* plants. *Nicotiana benthamiana* seeds were sown in commercial soil (from Compo Sana) and plants were grown for 8–9 weeks before performing transient expression.

### Preparation of constructs

All constructs were prepared using the gateway cloning method (Invitrogen). The plasmid pK7FWG2 was used for the preparation of *AtPHOT2*–GFP (green fluorescent protein) and *AtCLC2*–GFP via Phot2FPg, Phot2RPg, CLC2FPg, and CLC2RPg. Plasmid pK7WGF2 was used for the preparation of GFP–*AtPHOT2* via GPhot2FPg and GPhot2RPg. For the preparation of bimolecular fluorescence complementation (BiFC) constructs (*PHOT2*–NtermGFP, *CLC2*–NtermGFP), (*PHOT2*–CtermGFP, *CLC2*–CtermGFP), (NtermGFP–*AP2μ*) and (CtermGFP–*APμ*), pH7m34GW, pK7m34GW, pH7m24GW2, and pK7m24GW2 vectors were used, respectively. *AtERD2*–mCherry, mCherry–*SYP21*, mCherry–*SYP61*, and mCherry–*RABE1d* were constructed by overlapping PCR with ERD2FPg, ERD2RPg, SYP21FPg, SYP21RPg, SYP61FPg, SYP61RPg, RABE1dFPg, RABE1dRPg, mCherryNFP, mCherryNRP, mCherryCFP, and mCherryCRP. Five extra amino acids coding for glycine were added at the end of the first fusion gene RP to provide a proper folding environment to both the proteins. The final construct was transferred to pK7FWG2. Plasmids containing mCherry–*PIP1* and mCherry–*ARA7* (waveline138 and 2, respectively) were obtained from the NASC. They were originally reported by Geldner *et al.* (2009). The plasmid containing mCherry–*PIP1* was directly introduced into GV3101 *Agrobacterium* competent cells. The mCherry–*ARA7* fusion construct was transferred to a 35S promoter-containing plasmid pK7FWG2 using ARA7FPg and ARA7RPg. Plasmid pMDC7 was used to express the *PHOT2* gene under a β-oestradiol-inducible promoter (*AtPHOT2I*). The coding regions of *CLC2*, *ERD2*, *PHOT2*, *SYP21*, *SYP61*, and *RABE1d* were obtained from *Arabidopsis* cDNA. The cDNA was prepared from RNA isolated from *Arabidopsis* leaves. For the preparation of *PHOT2* ^*(D720/N)*^–GFP (aspartic acid at position 720 was changed to asparagine), site-directed mutagenesis was used to introduce the point mutation.

All final constructs were introduced into the *Agrobacterium* strain C58 unless mentioned otherwise. Details of plasmids and primers used can be found in Supplementary Table S1 available at *JXB* online.

### Transient expression and isolation of protoplasts

*Agrobacterium* constructs were grown at 28 °C for 1 d with constant shaking (200rpm). The culture was centrifuged and the pellet was suspended in an infiltration solution (10mM MES, 10mM MgCl_2_, and 100 μM acetosyringone). The final OD_600_ was maintained at 0.5 and the solution was kept at room temperature for at least 2h. After incubation, the solution was infiltrated into the abaxial side of *N. benthamiana* leaves. The expression was checked after 3–4 d using confocal microscopy. For the BiFC assay, co-expression, co-localization, and co-immunoprecipitation studies, the bacterial cultures were mixed in a 1:1 ratio (BiFC, Phot2–NtermGFP+CLC2–CtermGFP, Phot2–CtermGFP+CLC2–NtermGFP, Phot2–NtermGFP+CtermGFP–AP2μ, Phot2–CtermGFP+NtermGFP–AP2μ; co-localization/co-expression, Phot2–GFP+ERD2–mCherry/mCherry–SYP21/mCherry–SYP61/mCherry–ARA7/mCherry–RABE1d/mCherry–PIP1; co-immunoprecipitation, Phot2I+CLC2–GFP/GFP/GFP-AP2μ) before incubating them for infiltration.

For protoplast isolation, on the third day after infiltration, leaf discs were incubated in a protoplast enzyme solution (mannitol 500mM, CaCl_2_ 10mM, MES/KOH 5mM, cellulase 3%, and maceroenzyme 0.75%) for 10–12h. After incubation, the solution was passed through a nylon mesh. The protoplasts obtained were centrifuged and washed twice in the above solution without enzymes.

### Inhibitor treatment

For inhibitor treatment, leaves were infiltrated on the abaxial side using a needleless syringe and then incubated in an inhibitor solution [10 μM brefeldin A (BFA; Sigma), 50–200 μM tyrphostin23 (Sigma), 10 μM wortmannin (Sigma)] for 30/60min, respectively, before microscopic examination. As a control, water with 0.5% methanol/dimethylsulphoxide (DMSO) was infiltrated into leaves.

### Protein fraction preparation for western blotting

*phot2 localization studies Arabidopsis phot1* leaves and *Nicotiana* leaves (transiently expressing Atphot2–GFP) were detached and dark adapted for 12–14h. The dark-adapted leaves were either directly collected or irradiated with strong blue light (120 μmol m^–2^ s^–1^) for 30min before collection.

*phot2 turnover studies* Five- to six-week-old *A. thaliana phot1* mutants grown under *in vitro* conditions were dark-adapted for 12h and then transferred to 0.5% agar containing 0.5mM cycloheximide. The plants were incubated on cycloheximide plates for 1h and then either kept in the dark or illuminated with weak blue light (2 μmol m^–2^ s^–1^) or strong blue light (120 μmol m^–2^ s^–1^). Leaves were collected at 0, 60, 120, 240, and 360min.

*Nicotiana benthamiana* leaves transiently expressing Atphot2I were incubated in 5 μM oestradiol solution for 12h. The leaves were then transferred to sterile double-distilled water and kept in the growth chamber for the next 36h. After incubation, leaves were irradiated with strong blue light (120 μmol m^–2^ s^–1^). The leaves were collected at 0, 120, 240, and 360min after irradiation.

All samples collected were frozen in liquid nitrogen and then ground in a protein extraction buffer [TRIS-MES 50mM, sucrose 300mM, NaCl 150mM, CH_3_CO_2_K 10mM, EDTA 5mM, phenylmethylsulphonyl fluoride (PMSF) 1mM, and proteinase inhibitor cocktail]. The extract was centrifuged at 10 000 *g* for 10min and the supernatant was further centrifuged at 100 000 *g* for 75min. The supernatant obtained after the final centrifugation was stored as a cytosolic protein fraction. The pellet was re-suspended in a minimal amount of extraction buffer and stored as a microsomal protein fraction. The fractions were kept at –80 °C until use. The protein concentration in the fractions was determined using Lowry’s method. Blue light was obtained from light-emitting diodes with an emission maximum at 460nm.

### Co-immunoprecipitation and western blotting

*Nicotiana benthamiana* leaves were used for the transient expression of Atphot2I plus full-length–GFP/AtCLC2–GFP/GFP–AP2μ. Two days after infiltration, leaves were kept in 5 μM β-oestradiol solution for 12h to express phot2. After incubation, leaves were irradiated with strong blue light (120 μmol m^–2^ s^–1^) for 30min and frozen in liquid nitrogen. Microsomal fractions were isolated from the leaves. For immunoprecipitation, 50 μl of dynabeads–Protein G (Invitrogen) were taken and used according to the manufacturer’s instructions. In brief, phot2 antibody was added to the beads. The beads with the antibody were rotated top to bottom for 45min at room temperature. After rotation, the beads were gently washed and supplemented with the protein sample. The solution was rotated top to bottom for 3–4h at 4 °C. After incubation, the beads were again washed gently and finally suspended in SDS loading dye. The sample was heated at 100 °C for 8min and the protein sample was removed from the beads using a magnetic stand (Invitrogen). The isolated proteins were analysed by western blotting.

The primary antibodies used for western blotting were anti-Atphot2 (1:2000, custom made by Agrisera) and anti-GFP (1:4000, Clontech). Anti-Atphot2 antibody was raised in rabbits against short synthetic peptide from the N-terminal phot2 fragment. The peptide (NH_2_-)CSSKWMEFQDSAKIT(-CONH_2_) covered amino acids 54–67 of the protein. Final concentrations of the antibody preparations were optimized experimentally. The secondary antibodies were goat anti-rabbit IgG–horseradish peroxidase (HRP) (for phot2, 1:5000, Agrisera) and goat anti-mouse IgG–HRP (for GFP, 1:5000, Agrisera). ImageJ software was used to calculate the density of protein bands after western blotting.

### Confocal microscopy

All studies involving phot2–GFP and BiFC assay were performed on a Bio-Rad lasersharp2000 microscope. For phot2 imaging in *N. benthamiana* epidermal cells (expressing Atphot2–GFP), the cells were z-scanned at an interval of 1 μm using 10% laser (excitation in blue range 480nm). The first z-scan was always the 0min reading, after which the cells were continuously irradiated with blue light, and one more z-scan was performed after the irradiation. The last scan was the 5min reading. For protoplasts, a 3% laser was used and the other conditions were similar to those described for epidermal cells. For all studies, ×60 (oil immersion) magnification was used. For irradiation with blue light. a glass filter was used.

For all the co-localization studies a Nikon (NIS) or a Leica microscope was used. The images were taken at ×40/60 magnification (water/oil objective). The excitation/emission used for GFP was 488/525nm and for mCherry was 560/595nm.

## Results and Discussion

### Transiently expressed Atphot2–GFP shows blue-light-induced phot2 punctuate structures in the cytoplasm

**Fig. 1.**
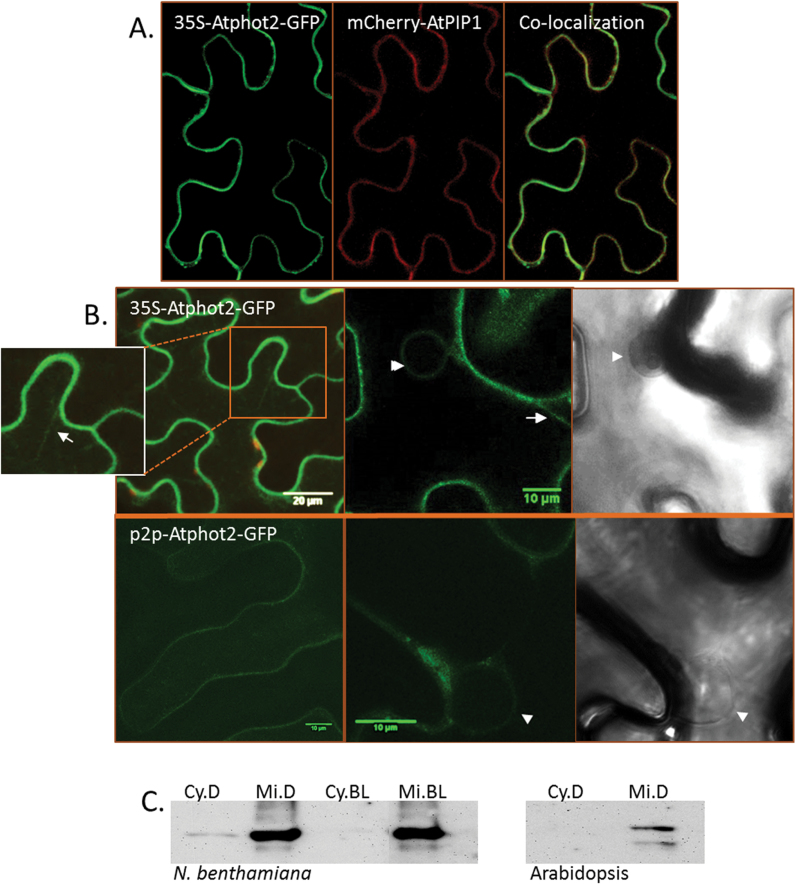
Phot2 localization. (A) Atphot2–GFP co-expressed with the plasma membrane marker PIP1 in *N. benthamiana*. (B) 35S-Atphot2–GFP and p2p-Atphot2–GFP showing green fluorescence at the plasma membrane, cytoplasmic strands (arrows), and nuclear envelope (arrowheads). (C) Western blot using anti-Atphot2 antibody. Cy, cytosolic; Mi, microsomal; D, dark; BL, blue light. The band corresponding to phot2 is visible in the cytosolic and microsomal fractions isolated from *N. benthamiana* leaves. In the *Arabidopsis* WT, phot2 is visible only in the microsomal fraction.

To determine the cellular localization, Atphot2–GFP (At5g58140) was transiently expressed in *N. benthamiana* under the 35S promoter or under its native promoter. The latter expression gave a visible but weaker green fluorescent signal compared with cells expressing 35S-phot2–GFP (Fig. 1B). Leaf samples dark adapted for 12–14h showed phot2–GFP at the plasma membrane. This was confirmed by co-localization with mCherry–PIP1 (Fig. 1A), the plasma membrane-localized aquaporin (Robinson *et al.*, 1996). Apart from the plasma membrane, cells expressing 35S-phot2–GFP showed fluorescence in cytoplasmic strands (Fig. 1B, arrows). Faint fluorescence was also visible in the cytosol. In addition, many cells showed phot2–GFP fluorescence (from both the 35S and native promoter) lining the nuclear envelope (Fig. 1B, arrowheads). The occurrence of cytosolic signal could be interpreted as soluble cytoplasmic phot2 or the receptor being present in intracellular compartment(s), for example the ER. Western blot analysis demonstrated phot2–GFP (expressed under the 35S promoter) in both microsomal and cytosolic soluble fractions isolated from dark-adapted *Nicotiana* leaves (Fig. 1C). In the cytosolic fraction, the band corresponding to phot2 had much lower intensity than that in the microsomal fraction. No phot2 was visible in the cytosolic fraction isolated from blue-light-irradiated *Nicotiana* leaves. The antibody used against phot2 was custom made by Agrisera and it is highly specific for *Arabidopsis*. It does not recognize any native protein from *N. benthamiana* (Supplementary Fig. S1 at *JXB* online). In *Arabidopsis* wild-type (WT) leaves phot2 was present only in the microsomal fraction and not in the cytosolic soluble fraction (Fig. 1C). This could be due to the weak natural expression of phot2 making it difficult to detect in the soluble fraction.

When GFP was fused to the N-terminus of phot2 (35S-GFP–phot2), the receptor was present at the plasma membrane, similar to phot2–GFP. However, additional strong fluorescent patches were seen in the cytoplasm, indicating hindrance to the proper localization of phot2 (Supplementary Fig. S2 at *JXB* online).

**Fig. 2.**
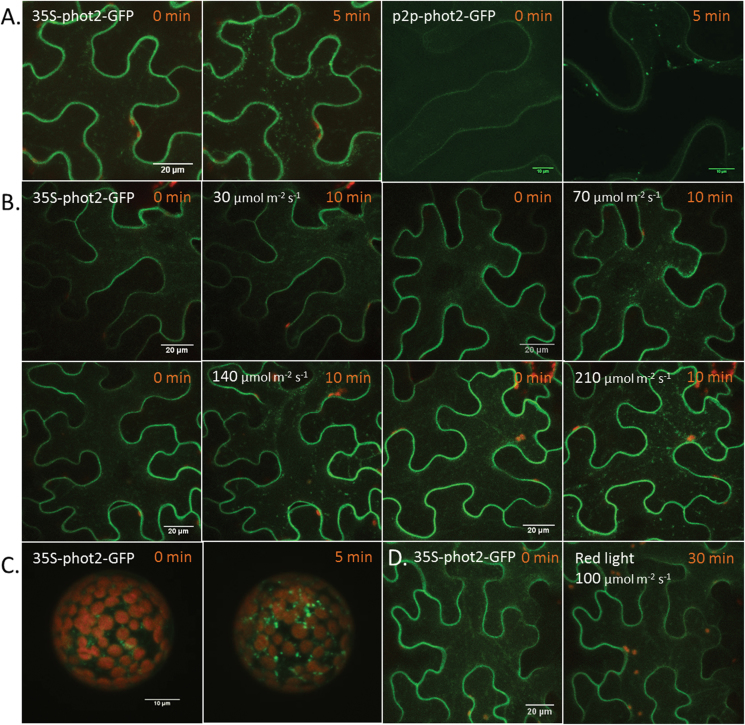
Blue-light-induced change in phot2 localization. (A–D) phot2–GFP transiently expressed in *N. benthamiana* leaves. (A–C) phot2–GFP showing blue-light-induced formation of punctuate structures in the cytoplasm: (A) blue laser (10%) irradiation for 5min; (B) blue (480nm) irradiation by filtered microscope light for 10min; (C) *Nicotiana* protoplasts irradiated with a blue laser (3%) for 5min; (D) *Nicotiana* epidermal cells irradiated with red light (650nm) by filtered microscope light for 30min. 0min represents images from the dark-adapted samples. p2p, phot2 native promoter.

The next step was to determine the localization of phot2 in the presence of blue light. Stably expressed Atphot2 forms cytoplasmic punctuate structures in response to blue light irradiation (Kong *et al.*, 2006, 2007; Aihara *et al.*, 2008). In the transient expression system used here, continuous irradiation with a blue laser beam or blue light from the microscope lamp caused phot2-labelled punctuate structures to appear in the cytoplasm (Fig. 2A, B) in all irradiated cells. Their number increased with increasing light intensity. After 10min of blue light irradiation, the number of phot2-labelled punctuate structures was much lower at 30 μmol m^–2^ s^–1^ than at 140 μmol m^–2^ s^–1^. In daylight, the blue light intensity ranges from 100 μmol m^–2^ s^–1^ to 150 μmol m^–2^ s^–1^ at most, showing that formation of phot2 punctuate structures occurs within minutes under natural conditions. The presence of similar phot2 punctuate structures in *N. benthamiana* mesophyll cell protoplasts (Fig. 2C) supported the results obtained with the epidermal cells. Phot2-labelled punctuate structures were absent in cells outside the irradiation area. Additionally, no punctuate structures were present in epidermal cells irradiated with red light (Fig. 2D). A 15min film showing the induction and rapid cytosolic movements of phot2-labelled punctuate structures can be seen in Supplementary Movie S1 at *JXB* online. Intriguingly, the presence of punctuate structures was not accompanied by any obvious decrease in the plasma membrane fluorescence shown by analysis of the GFP fluorescence intensity at the plasma membrane (Supplementary Fig. S3).

**Fig. 3.**
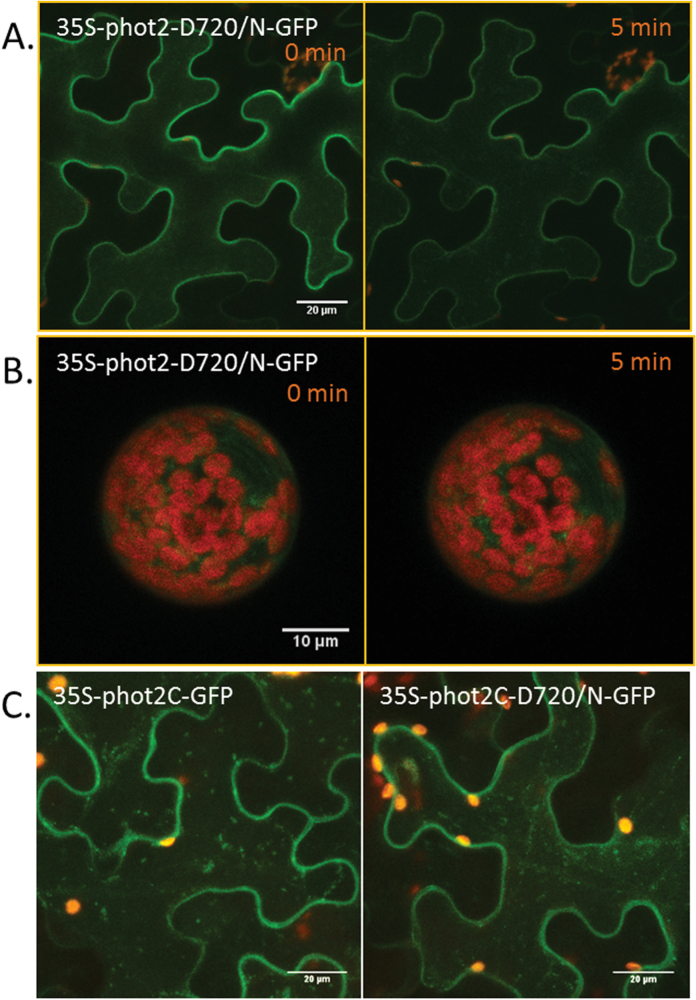
Transient expression and localization of the phot2 kinase domain. (A, B) Kinase-inactive phot2 (35S-phot2D720/N–GFP) transiently expressed in *N. benthamiana* epidermal cells and protoplasts. 0min represents images from the dark-adapted samples. (C) Kinase domain (534–915 amino acids, 35S-phot2C–GFP) or kinase-inactive domain (35S-phot2C-D720/N–GFP) transiently expressed in *Nicotiana* epidermal cells. All cells were irradiated with a blue laser beam which was also used for excitation of GFP.

Transient expression of the kinase-inactive 35S- phot2^(D720/N)^–GFP did not show blue-light-dependent formation of punctuate structures. Similar findings were also reported by Kong and co-workers (2006, 2007). The transiently expressed mutated phot2 was present at the plasma membrane and faintly in the cytoplasm (Fig. 3A, B), which means it had a similar localization to phot2-expressing dark-adapted samples (Fig. 1B). Experiments were then conducted to investigate whether blue-light-mediated phot2 autophosphorylation was required for the appearance of punctuate structures. The transient expression of kinase domains alone, either phot2-kinase–GFP (P2C, 534–915 amino acids) or phot2-kinase inactive D720/N–GFP (P2C-D720/N), led to green fluorescence at the plasma membrane and in cytosolic punctuate structures, irrespective of the presence/absence of light (Fig. 3C). The obtained results are noteworthy as they suggest that the blue-light-mediated formation of phot2 punctuate structures is not primarily due to receptor autophosphorylation. Blue light disrupts the interaction between the kinase and LOV2 domain, resulting in structural changes. Additionally, Pfeifer *et al.* (2010) showed that autophosphorylation of phot2 is not a prerequisite for light-induced structural changes. The results in the present study prove that phot2 trafficking is primarily due to conformational changes in the kinase domain when it separates from the LOV2 domain, and not to the receptor autophosphorylation.

The emerging punctuate structures could be due to the translocation of phot2 from the plasma membrane (internalization) or from the cytoplasm. As stated before, the presence of phot2-labelled punctuate structures was not accompanied by any significant decrease in plasma membrane fluorescence after blue light irradiation (Supplementary Fig S3 at *JXB* online). Additionally, in *N. benthamiana* leaves, phot2 levels decreased in the cytosolic soluble fraction after blue light irradiation (Fig. 1C). This implies that blue light might activate phot2 translocation from the cytoplasm. Co-localization studies were carried out to determine further features of the phot2 translocation pathway.

### In blue light phot2 is present in the Golgi and post-Golgi compartments

**Fig. 4.**
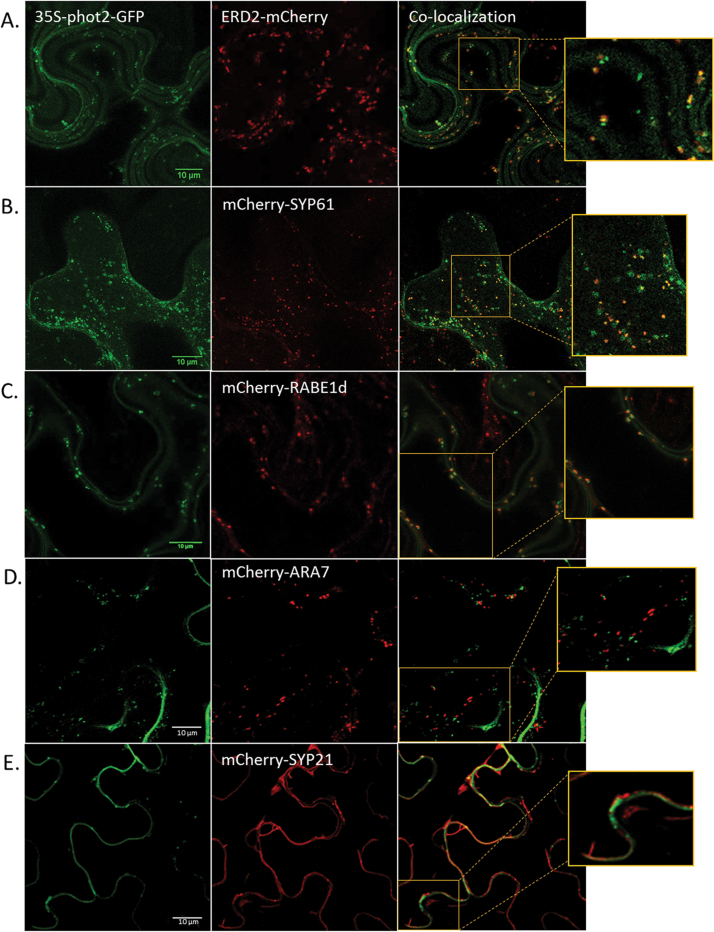
Co-localization studies using intracellular compartment markers. (A–E) 35S-phot2–GFP co-expressed with (A) the *cis*-Golgi marker ERD2l; (B) the *trans*-Golgi network marker SYP61; (C) the post-Golgi vesicle marker RABE1d; (D) the late endosome marker ARA7; and (E) the vacuolar membrane marker SYP21.

**Fig. 5.**
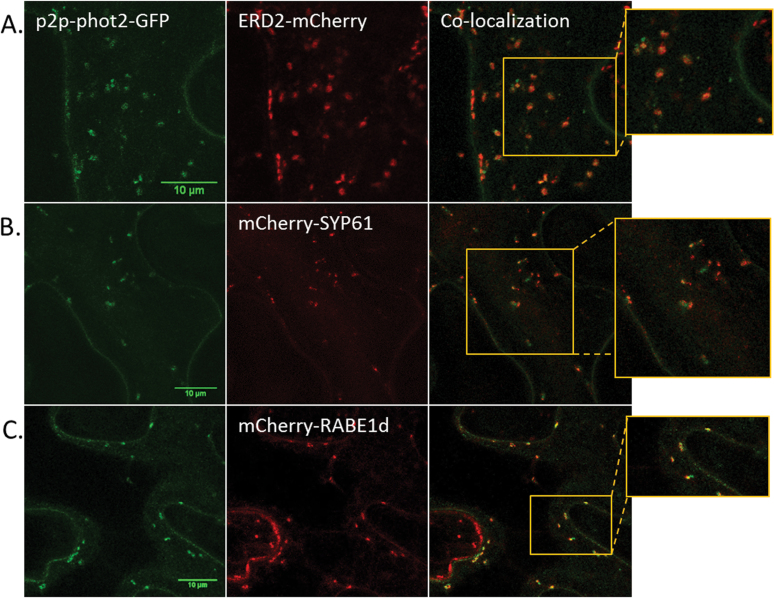
Co-localization studies using intracellular compartment markers. (A–C) p2p-phot2–GFP co-expressed with (A) the *cis*-Golgi marker ERD2; (B) the *trans*-Golgi network marker SYP61; and (C) the post-Golgi vesicle marker RABE1d.

In *Arabidopsis* cotyledon epidermal cells, phot2-labelled punctuate structures were shown to co-localize with the Golgi apparatus marker KAM1ΔC (Kong *et al.*, 2006). However, the route of phot2 translocation remained unknown. To identify this route, different cellular compartment markers fused with an mCherry fluorescent tag were used. Strong co-localization was observed between phot2 and the *cis*-Golgi marker AtERD2 (Figs 4A, 5A), confirming the Golgi localization of phot2 punctuate structures. Along with its Golgi localization, phot2 was also present in other cellular compartments. Phot2 showed co-localization with the *trans*-Golgi network (AtSYP61), and most of the localized structures were in close association with the Golgi complex (Figs 4B, 5B). Some phot2-labelled punctuate structures also co-localized with the Golgi/post-Golgi vesicle marker AtRABE1d (Figs 4C, 5C). The receptor was absent from late endosomes (AtARA7) and the vacuolar membrane (AtSYP21) (Fig. 4D, E). The plasma membrane can be distinguished from the tonoplast because the latter shows an uneven shape at the cell periphery as it bends inwards. The results indicate that phot2 travels not only to the Golgi apparatus but also to the *trans*-Golgi and other post-Golgi vesicles. The Manders coefficients for the co-localization studies are shown in Supplementary Fig. S4 at *JXB* online.

**Fig. 6.**
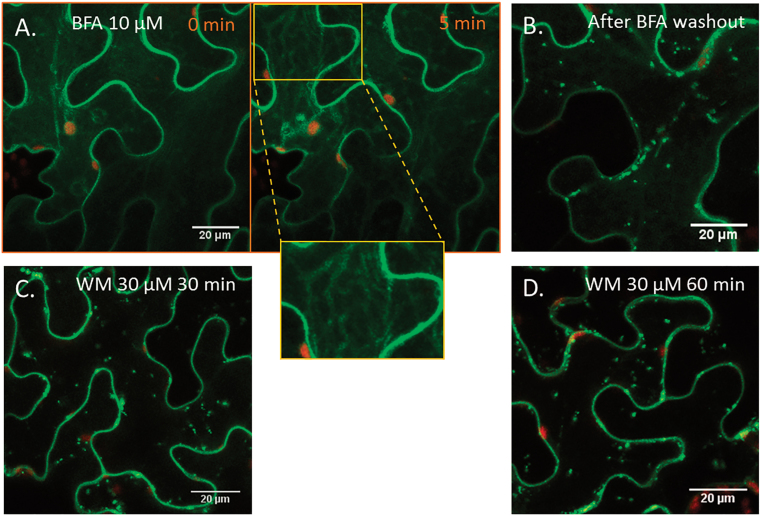
Effect of BFA and wortmannin (WM) on formation of phot2-labelled punctuate structures. (A–D) *Nicotiana* epidermal cells expressing 35S-phot2–GFP. (A) In the presence of 10 μM BFA, a criss-cross pattern was visible in the epidermal cells after irradiation with a blue laser (10%) for 5min. (B) Removal of BFA restored the formation of phot2 punctuate structures after blue light irradiation. (C, D) Application of WM had no inhibitory effect on blue-light-induced phot2 punctuate structures.

The above findings were confirmed by the application of pharmacological agents. The chemical BFA causes a disassembly of the Golgi complex thereby blocking the secretory pathway. BFA effects are tissue specific in plants and thus there are non-uniform responses depending on the cell type. In the epidermal cells of *Arabidopsis* leaves, BFA causes reabsorption of the Golgi membranes into the ER (Robinson *et al.*, 2008). In the present study, BFA disrupted the formation of phot2 punctuate structures in *N. benthamiana* epidermal cells. Upon blue light, a distinctive criss-cross fluorescent pattern became visible in most cells, indicating the presence of phot2 in the modified ER–Golgi complex (Fig. 6A). BFA effects are reversible, and removal of the chemical quickly re-establishes the Golgi apparatus (Robinson *et al.*, 2008). Washing out BFA restored the formation of phot2 punctuate structures (Fig. 6B). Unlike BFA, wortmannin had no inhibitory effect (Fig. 6C, D). The chemical impairs vacuolar trafficking by the fusion and swelling of multivesicular bodies (Takac *et al.*, 2012) and also suppresses the internalization of proteins from the plasma membrane (Ito *et al.*, 2012).

**Fig. 7.**
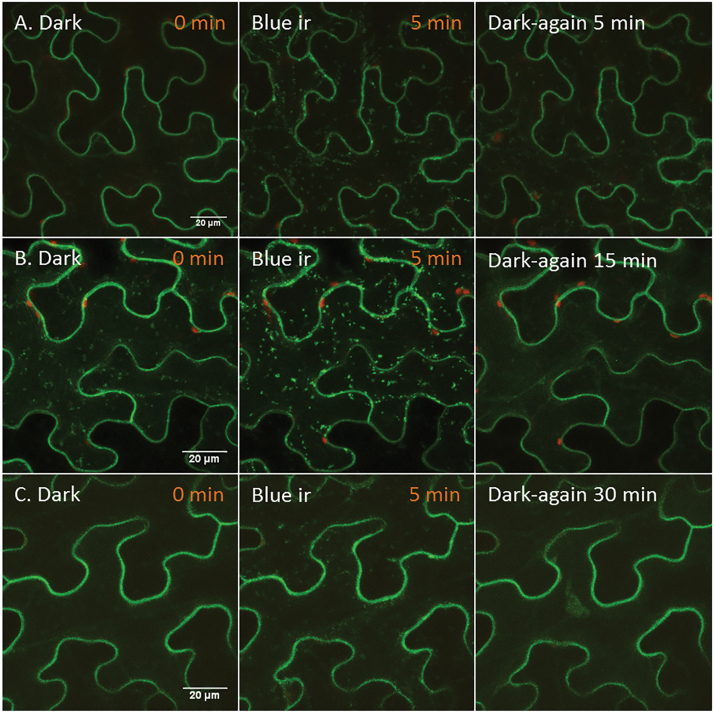
Time required for disappearance of phot2-labelled punctuate structures after blue light irradiation. (A–C) Dark-adapted *Nicotiana* cells (transiently expressing 35S-phot2–GFP) were irradiated with a blue laser (10%) for 5min and the samples were kept in darkness again for the specified period. After dark incubation, one more z-scan was performed to see the localization of phot2. 0min represents images from the dark-adapted samples. ir, irradiation.

The absence of any effect of wortmannin on phot2 trafficking supports the contention that upon irradiation with blue light phot2 is transferred from the cytoplasm to the Golgi apparatus and then to the post-Golgi vesicles. The presence of phot2 in this route could suggest that the receptor targets the plasma membrane. Direct evidence of secretory cargo passing through the *trans*-Golgi network is limited. However, confocal laser scanning microscopy analysis after inducible expression of secretory GFP and BRI1–yellow fluorescent protein as well as immunogold electron microscopy of a xyloglucan epitope confirmed that at least some secretory cargo molecules pass through the *trans*-Golgi network to reach the plasma membrane (Viotti *et al.*, 2010). Another possibility could be that phot2 continuously cycles between the cytoplasm, Golgi, post-Golgi vesicles, and plasma membrane in darkness. Upon irradiation with blue light, phot2 travels from the cytoplasm and is trapped in the Golgi and post-Golgi structures. The Golgi translocation of phot2 might be a mechanism to regulate the density of phot2 at the anticlinal and periclinal walls, potentially of key importance for chloroplast responses to light. Interestingly, the phot2-labelled punctuate structures which appeared after irradiation with blue laser (5min) disappeared from the cytoplasm 15–30min after the samples were placed in darkness again (Fig. 7). In darkness, phot2 quickly dephosphorylates (Tseng and Briggs, 2010). This dephosphorylated phot2 can be translocated by a forward (towards the cell periphery) and/or backward (towards the cytoplasm) pathway. The fate of dephosphorylated phot2 needs further examination.

### Clathrin associates with phot2 but does not participate in formation of punctuate structures

Clathrin subunits are three-legged triskelions (each leg composed of a heavy and a light chain) which interact with each other along with adaptor and accessory proteins. Together the complex is responsible for the internalization of plasma membrane proteins (clathrin-mediated endocytosis) and for the targeting of proteins from the Golgi to the cell membrane (secretory pathway/exocytosis) (Jaiswal *et al.*, 2009; Chen *et al.*, 2011). Using transgenic *Arabidopsis* seedlings expressing phot1- and phot2–GFP, Kaiserli *et al.* (2009) reported the presence of a clathrin heavy chain (CHC) in immunoprecipitates of phot1 and phot2. It was decided to examine the involvement of clathrin in phot2 translocation in *N. benthamiana*.

**Fig. 8.**
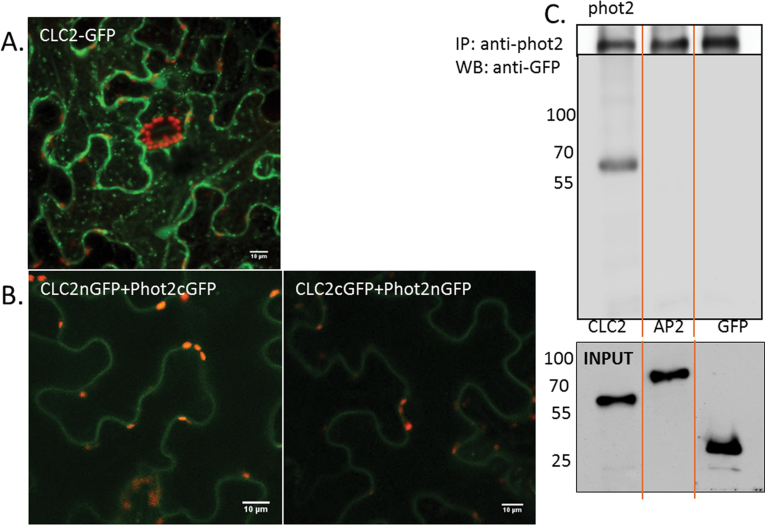
Phot2 association with light chain2 of clathrin. (A) Light chain2 of clathrin (AtCLC2) transiently expressed in *N. benthamiana.* (B) BiFC technique used to examine the association between phot2 and CLC2. (C) Co-immunoprecipitation assay: the microsomal fraction proteins isolated from *N. benthamiana* leaves were subjected to immunoprecipitation (using Atphot2 antibody) and then to western blot. The upper blot shows phot2 (after use of an anti-Atphot2 antibody) in *Nicotiana* leaves transiently expressing phot2I plus CLC2–GFP, GFP–AP2μ, and GFP respectively. The lower blot shows a single band corresponding to CLC2–GFP after anti-GFP staining. As control (input), microsomal fractions (100 μg) were also immunoblotted before immunoprecipitation; the bands were identified using anti-GFP antibody. Numbers represent molecular weights (kDa) of the protein marker (Thermo Scientific).

AtCLC2 (clathrin light chain 2, At2g40060) interacts with the C-terminus of mammalian CHC and with AtCHC1 (Scheele and Holstein, 2002; Damme *et al.*, 2011). In addition, AtCLC2 and dynamin-related proteins are present in the dynamic foci at the cell cortex (Konopka *et al.*, 2008; Fujimoto *et al.*, 2010) and participate in clathrin-mediated membrane dynamics. In agreement with previous studies (Konopka *et al.*, 2008; Damme *et al.*, 2011), AtCLC2–GFP was found at the plasma membrane and intracellular compartments (Fig. 8A). A BiFC assay showed an association between AtCLC2 and phot2 in *Nicotiana* leaves. With the combination CLC2–CtermGFP+Phot2–NtermGFP and CLC2–NtermGFP+Phot2–CtermGFP, a green fluorescent signal was observed at the plasma membrane (Fig. 8B). The obtained signal was not very strong but was clearly visible under the microscope. The controls for the BiFC study are shown in Supplementary Fig. S5 at *JXB* online. The findings were further confirmed using co-immunoprecipitation. A single band corresponding to AtCLC2–GFP (~60kDa) co-precipitated with phot2 (Fig. 8C).

**Fig. 9.**
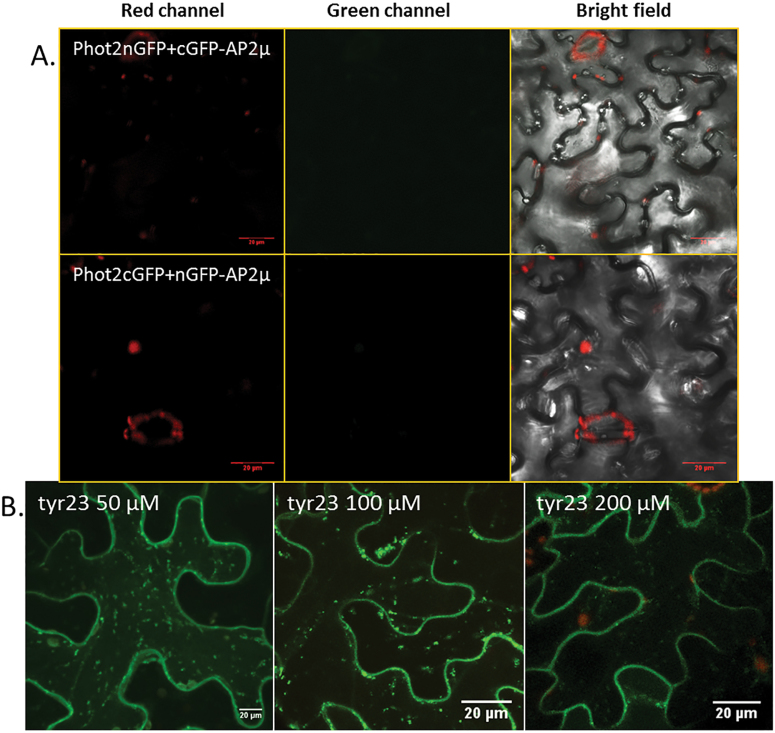
Phot2 association with AP2μ and the effect of tyrphostin23 (tyr23) on phot2 translocation. (A) Images from the BiFC study showing no association between phot2 and AP2μ. (B) *Nicotiana* epidermal cells expressing 35S-phot2–GFP. Phot2 localization was examined after irradiation with blue light in cells pre-treated with 50, 100, and 200 μM tyr23.

Genetic and chemical tools were used to check if the association of phot2 with clathrin is involved with the formation of punctuate structures. Clathrin does not bind to the membrane directly; its binding depends on many accessory and adaptor proteins. One of the adaptor proteins which directly interact with the receptor molecule at the plasma membrane is AP2 (adaptor protein 2). In mammalian cells, it is the μ2 subunit of AP2 which recognizes and binds to the tyrosine-based signals YXXΦ (Φ represents a bulky hydrophobic residue and X can be any amino acid) in plasma membrane proteins (Ohno *et al.*, 1995). In plants, the AtAP2μ (At5g46630) subunit interacts with the defence receptor LeEix2 and this interaction is required for the receptor internalization (Bar *et al.*, 2009). Recent work by Di Rubbo *et al.* (2013) also showed the involvement of AP2 in the endocytosis of the brassinosteroid receptor BRI1. In the present study, no interaction was observed between phot2 and AtAP2μ (At5g46630) by the use of the BiFC assay and co-immunoprecipitation (Fig. 8C, 9A). However, an AP2 subunit other than At5g46630 might be interacting with phot2. To understand further the involvement of clathrin, a chemical approach was used. Among the chemicals, a structural analogue of tyrosine, tyrphostin23, binds to the μ2 subunit of the AP2 adaptor complex and blocks its interaction with the tyrosine motif on the membrane receptors (Banbury *et al.*, 2003). The drug inhibits the internalization of FM4-64 in BY-2 cells (Lam *et al.*, 2009), of the human transferrin receptor in *Arabidopsis* protoplasts (Ortiz-Zapater *et al.*, 2006), and of PIN2 and IRT1 in *Arabidopsis* roots (Dhonukshe *et al.*, 2007; Barberon *et al.*, 2011). The optimal concentration (50–100 μM) of tyrphostin23 had no inhibitory effect on the formation of blue-light-mediated phot2 punctuate structures (Fig. 9B). Only at the high concentration of 200 μM was the appearance of punctuate structures suppressed to a certain extent (Fig. 9B). This effect was irreversible, indicating inhibitor-induced damage to the cells at high concentration. These results confirm that clathrin does not participate in the formation of phot2-labelled punctuate structures. Clathrin associated with phot2 might be involved in the secretory pathway. Jaiswal and co-workers (2009) showed that post-Golgi vesicle-mediated exocytosis is regulated by clathrin and dynamin. However, the absence of phot2–CLC2 fluorescence in the punctuate structures rules out this possibility. Another possible involvement of clathrin might be in the ubiquitination-dependent degradation of the receptor as suggested for phot1 (Kaiserli *et al.*, 2009; Roberts *et al.*, 2011). To examine this question, the degradation rate of the receptor in darkness and upon blue irradiation was checked.

### phot2 is continuously degraded in darkness

**Fig. 10.**
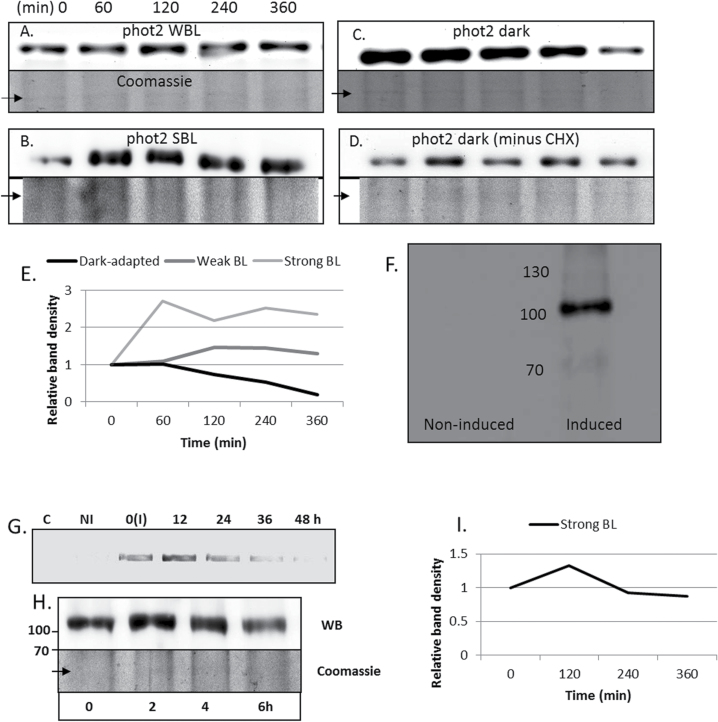
Phot2 turnover. (A–C) Phot2 detected with an anti-Atphot2 antibody in microsomal protein fractions (50 μg) isolated from leaves of the *Arabidopsis phot1* line [pre-treated with cycloheximide (CHX)] (A) after weak blue light (WBL; 2 μmol m^–2^ s^–1^), (B) after strong blue light (SBL; 120 μmol m^–2^ s^–1^) irradiation, and (C) in dark-adapted plants. (D) Phot2 detected by immunoblotting in dark-adapted leaf samples not pre-treated with CHX. The upper picture represents the western blot and the lower is the Coomassie blue staining. (E) Quantitative analysis of phot2 turnover in *Arabidopsis phot1* mutant leaves. (F) Western blot analysis of microsomal protein fractions (100 μg) obtained from *N. benthamiana* leaves transiently expressing phot2I. No phot2 signal was detected after anti-Atphot2 staining under non-inducible conditions (left column) while a phot2 signal appeared after induction with a 5 μM β-oestradiol for 12h (right column). The molecular weight (kDa) of the protein marker can be seen in the centre. (G) mRNA levels of phot2 in *N. benthamiana* leaves transiently expressing phot2I. 0(I) represents mRNA levels immediately after 12h induction with a 5 μM β-oestradiol solution. (H) Protein levels of *PHOT2* in *N. benthamiana* leaves transiently expressing phot2I. At 36h after induction, the leaf samples were continuously irradiated with strong blue light and the samples were collected every 2h. The concentration of the protein used was 50 μg. (I) Quantitative analysis of phot2 turnover in *N. benthamiana*. C, control; NI, non-induced; I, induced. All studies were performed at least three times on independent samples.

The western blot technique was applied to study the phot2 turnover in darkness and blue light. Cycloheximide was used as a protein synthesis inhibitor to block the formation of new phot2. In darkness, *Arabidopsis phot1* mutant leaves showed a gradual decrease in the amount of phot2 (Fig. 10C). The half-life calculated was 3–4h (Fig. 10E). In the absence of cycloheximide, the levels of phot2 remained constant (Fig. 10D), pointing to the continuous synthesis of the receptor. In contrast, no obvious changes were observed in the levels of phot2 in the presence of weak or strong blue light, even after 6h of continuous irradiation (Fig. 10A, B, E).

An additional experiment was carried out to exclude the possibility that the constant level of phot2 under blue light was due to degradation of cycloheximide by light. The phot2 protein turnover was examined in *N. benthamiana* leaves transiently expressing phot2I. A full and strong expression of phot2 was obtained after 12h induction in a β-oestradiol solution, while phot2 was absent under non-inducing conditions (Fig. 10F). At 36h after the induction, the Atphot2 mRNA levels were almost non-existent in the *N. benthamiana* leaves (Fig. 10G), which indicated that no new phot2 protein was synthesized. Thus, it seemed the right time to perform the turnover test. The western blot analysis of the microsomal fractions (obtained from leaves irradiated with strong blue light from 36h to 42h) showed a slow degradation of phot2 (Fig. 10H, I).

As stated earlier, Roberts and co-workers (2011) demonstrated phot1 ubiquitination in the presence of blue light. Likewise, phot2 seems to degrade both in darkness and in blue light, the latter at a slower rate. The association of phot2 with clathrin might be involved in this mechanism. The molecular mechanism and transport route involved in the process need to be examined in future.

### Concluding remarks

Phot1 and phot2 are plasma membrane-associated blue light receptor kinases. In the presence of blue light, phot1 translocates to the cytoplasm and phot2 to the Golgi complex. In this work, an attempt was made to obtain a deeper insight into phot2 translocation.

Transient expression is a good system for studying phot2 trafficking in plants. Atphot2–GFP transiently expressed under 35S or its native promoter in *N. benthamiana* behaves in a similar way to stably expressed phot2–GFP in *Arabidopsis*. In darkness, phot2 is present at the plasma membrane and in the cytoplasm. Blue light activates the movement of phot2 from the cytoplasm into discreet punctuate structures. The kinase domain of phot2 but not the blue-light-mediated autophosphorylation is required for the formation of punctuate structures. Intracellular compartment markers showed that phot2 moves to the Golgi complex, *trans*-Golgi network, and post-Golgi vesicles, probably taking the secretory route. On the other hand, like most membrane receptors, phot2 also undergoes degradation in both darkness and blue light. The route for phot2 degradation is different from phot2 translocation to the Golgi. Clathrin units might be involved in the degradation route. In brief, phot2 seems to follow two routes, one from the cytoplasm to the Golgi complex and the other from the plasma membrane to degradation. The significance of the phot2 localization to the Golgi complex and post-Golgi vesicles is unclear. The strong blue-light-mediated chloroplast avoidance response is controlled exclusively by phot2 (Banas *et al.*, 2012). However, BFA, which disturbs the transfer of phot2 to the Golgi, had no inhibitory effect on chloroplast movements in *Arabidopsis* (data not shown).

The new findings give us a better understanding of phot2 trafficking in blue light. The results also raise further questions, which need to be addressed in future. What is the route of phot2-labelled punctuate structures upon the switching off of blue light? How is phot2 degraded? Is the mechanism similar to phot1 ubiquitin-dependent degradation? What role does phot2 translocation play in receptor signalling?

## Supplementary data

Supplementary data are available at *JXB* online.

Figure S1. Anti-Atphot2 antibody specificity.

Figure S2. 35S-GFP–phot2 transiently expressed in *N. benthamiana* epidermal cells.

Figure S3. Plasma membrane fluorescence intensity before and after blue light irradiation.

Figure S4. Manders coefficients for co-localization studies.

Figure S5. Controls for the BiFC technique.

Movie S1. Blue-light-induced phot2 translocation and movement of phot2-labelled punctuate structures.

Table S1. Plasmids and primers used in the study.

Supplementary Data

## Abbreviations:

BFA: brefeldin A
BiFC: bimolecular fluorescence complementation
CHC: clathrin heavy chain
CLC: clathrin light chain
ER: endoplasmic reticulum
phot: phototropin
WT: wild type.
