# Supplementary material for: Blue-light-activated phototropin2 trafficking from the cytoplasm to Golgi/post-Golgi vesicles
Source: J Exp Bot. 2014 May 12;65(12):3263–76. doi: 10.1093/jxb/eru172 (PMC4071840; doi:10.1093/jxb/eru172)
Supplement: Supplementary Data [file supp_65_12_3263__index.html]

Blue-light-activated phototropin2 trafficking from the cytoplasm to Golgi/post-Golgi vesicles — Blue-light-activated phototropin2 trafficking from the cytoplasm to Golgi/post-Golgi vesicles — Supplementary Data 

# Blue-light-activated phototropin2 trafficking from the cytoplasm to Golgi/post-Golgi vesicles

## Supplementary Data

Data files

**Files in this Data Supplement:**

- Supplementary Data - Supplementary Data
- Supplementary Data - Supplementary Data
