# Supplementary material for: Blue-light-activated phototropin2 trafficking from the cytoplasm to Golgi/post-Golgi vesicles
Source: J Exp Bot. 2014 May 12;65(12):3263–76. doi: 10.1093/jxb/eru172 (PMC4071840; doi:10.1093/jxb/eru172)
Supplement: Supplementary Data [file supp_eru172_jexbot117341_file001.pdf]

Supplemental file

Figure S1: Anti-Atphot2 antibody specificity.

Total protein fraction was isolated from leaves of *Arabidopsis thaliana* (WT) and *Nicotiana benthamiana* and subjected to western blotting using Atphot2 antibody. Phot2 protein band is visible only in Arabidopsis. Plants were either dark-adapted for 12 h or kept under white light in growth chambers for 3 h before the protein extraction. 100 µg protein sample was loaded in each lane.

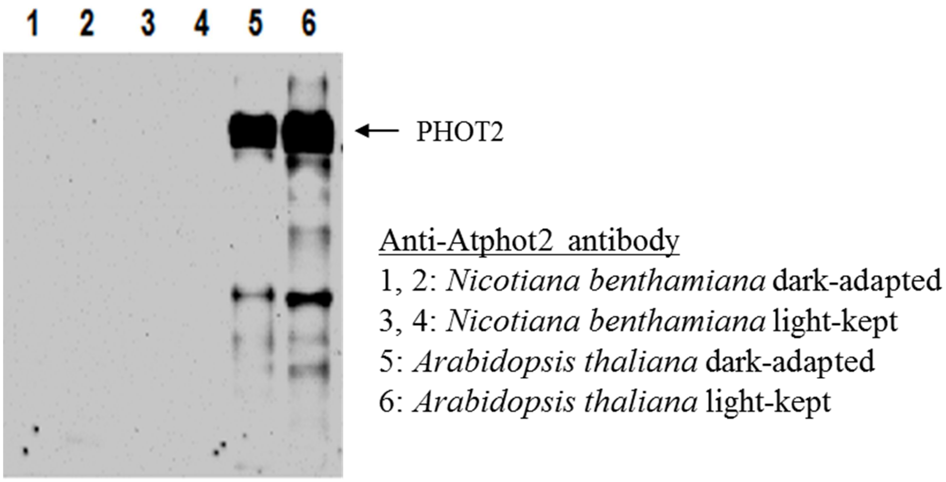

Anti-Atphot2 antibody
1, 2: *Nicotiana benthamiana* dark-adapted
3, 4: *Nicotiana benthamiana* light-kept
5: *Arabidopsis thaliana* dark-adapted
6: *Arabidopsis thaliana* light-kept

Figure S2: 35S-GFP-phot2 transiently expressed in *N. benthamiana* epidermal cells. Arrows are showing clumping of phot2 in the cytoplasm.

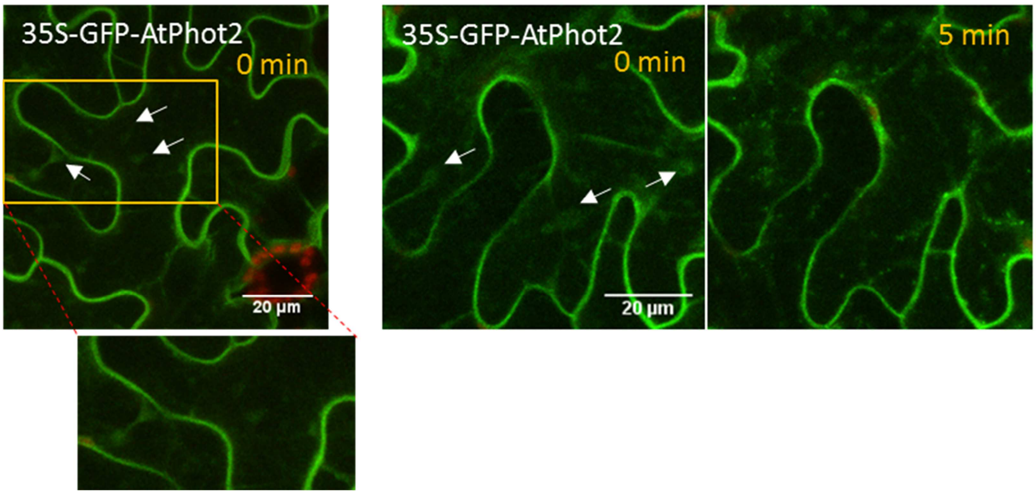

<u>Figure S3</u>: Plasma membrane fluorescence intensity before and after blue-light irradiation. The corrected total fluorescence is calculated by subtracting the background fluorescence. Ten different cells were examined in each case.

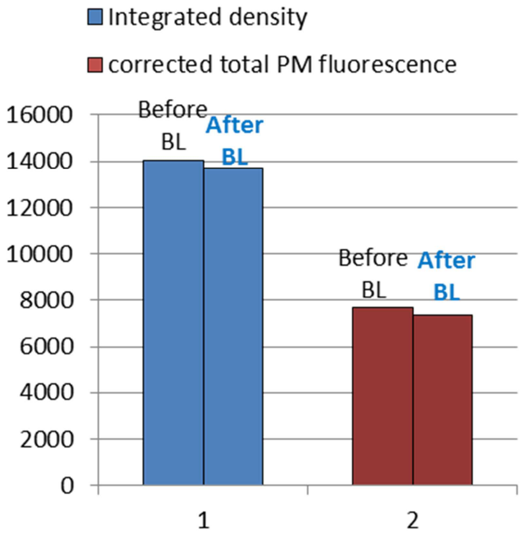

<u>Figure S4</u>: Manders coefficient for co-localization studies (calculated using ImageJ plugin JACop).

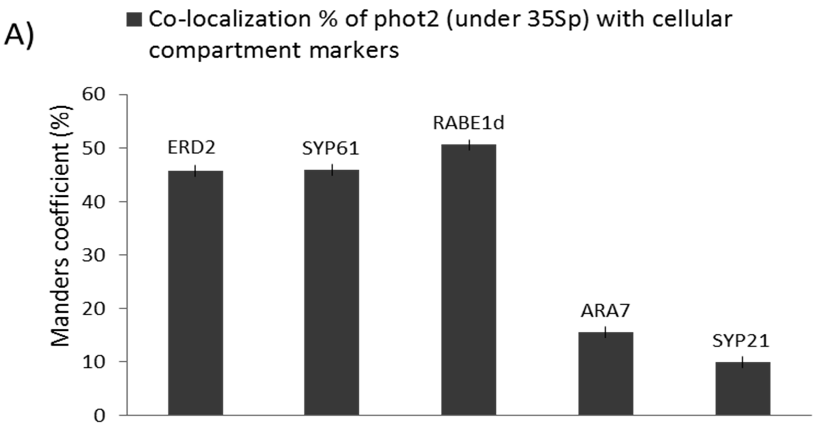

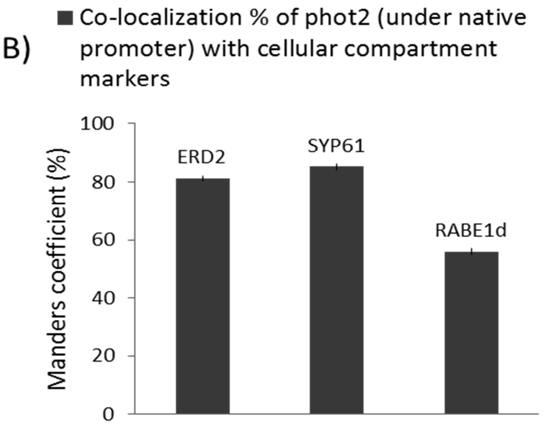

Figure S5: Controls of BiFC technique.

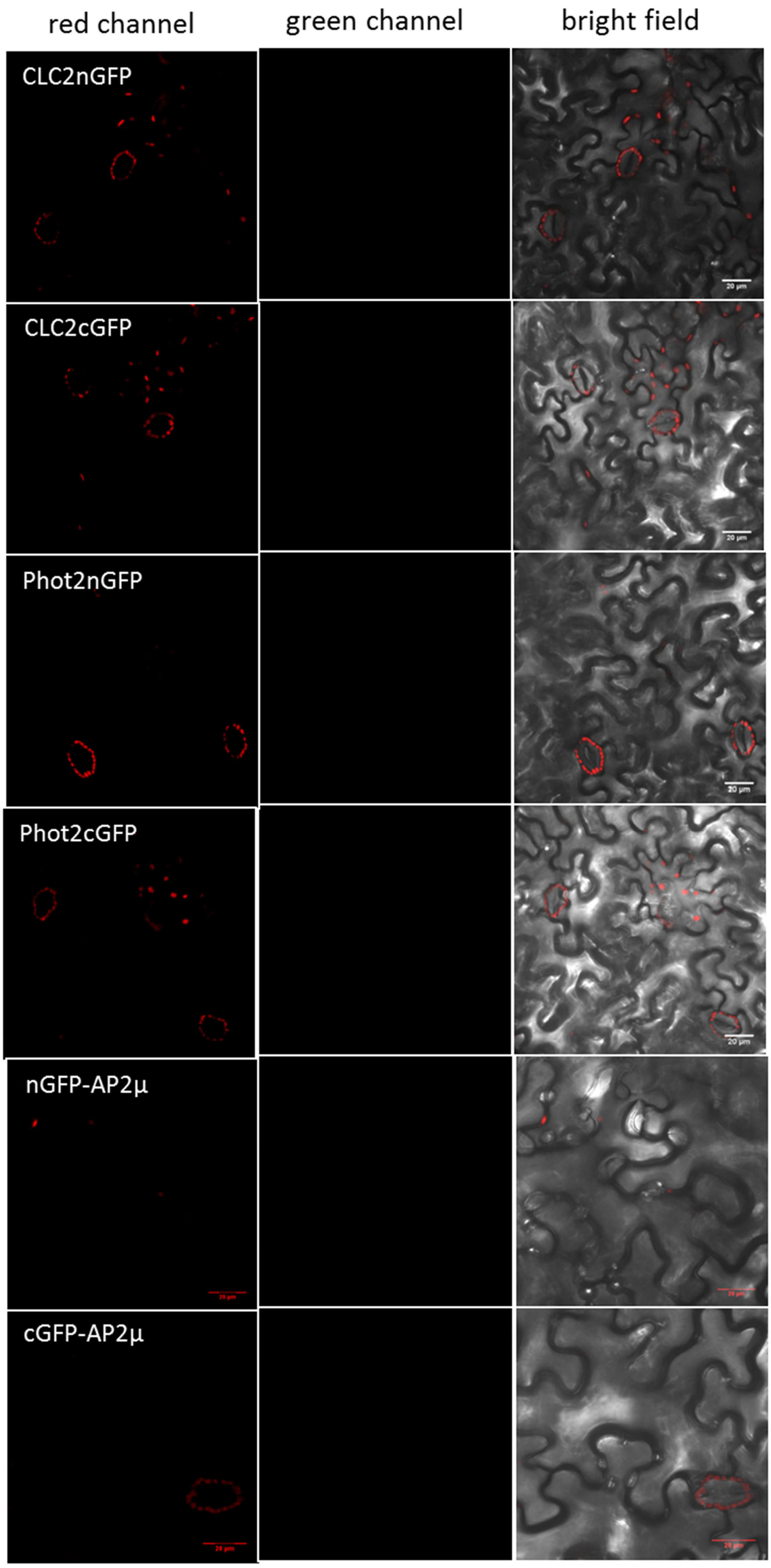

Table S1: Plasmids and primers used in the study

PLASMIDS	
pK7FWG2	Gateway destination vector for expression of the gene under 35S promoter with C-terminal GFP fusion
pK7WGF2	Gateway destination vector for expression of the gene under 35S promoter with N-terminal GFP fusion
pH7m34GW	Gateway destination vector for expression of the gene under 35S promoter with N-terminal part of GFP at C-terminal of protein
pK7m34GW	Gateway destination vector for expression of the gene under 35S promoter with C-terminal part of GFP at C-terminal of protein
pK7m24GW2	Gateway destination vector for expression of the gene under 35S promoter with C-terminal part of GFP at N-terminal of protein
pH7m24GW2	Gateway destination vector for expression of the gene under 35S promoter with N-terminal part of GFP at N-terminal of protein
pMDC7	Gateway destination vector for expression of the gene under β-estradiol inducible promoter.

GATEWAY TECHNOLOGY (PRIMERS)	
ARA7FPg	GGGGACAAGTTTGTACAAAAAAGCAGGCTCCATGTTGAGCAAGGGCGAGG
ARA7RPg	GGGGACCACTTTGTACAAGAAAGCTGGGTCCTAAGCACAACAAGATGAGCTC
CLC2FPg	GGGGACAAGTTTGTACAAAAAAGCAGGCTCCATGTCTGCCTTTGAAGACGATTC
CLC2RPg	GGGGACCACTTTGTACAAGAAAGCTGGGTCAGCAGCAGTAACTGCCTCAG
ERD2FPg	GGGGACAAGTTTGTACAAAAAAGCAGGCTCCATGAATATCTTTAGATTTGCTGGCG
ERD2RPg	CATACCTCCACCTCCACCAGCCGGAAGCTTAAGTTTGG
mCherryCFPg	GGTGGAGGTGGAGGTATGGTGAGCAAGGGCGAG
mCherryCRPg	GGGGACCACTTTGTACAAGAAAGCTGGGTCCTACTTGTACAGCTCGTCCATG
mCherryNFPg	GGGGACAAGTTTGTACAAAAAAGCAGGCTCCATGGTGAGCAAGGGCGAG
mCherryNRPg	CTTACCTCCACCTCCACCCTTGTACAGCTCGTCCATG
Phot2FPg	GGGGACAAGTTTGTACAAAAAAGCAGGCTCCATGGAGAGGCCAAGAGCCC
Phot2RPg	GGGGACCACTTTGTACAAGAAAGCTGGGTCGAAGAGGTCAATGTCCAAGTCC
GPhot2FPg	GGGGACAAGTTTGTACAAAAAAGCAGGCTCCATGGAGAGGCCAAGAGCCC
GPhot2RPg	GGGGACCACTTTGTACAAGAAAGCTGGGTCGAGGTCAATGTCCAAGTCC
Phot2stopRPg	GGGGACCACTTTGTACAAGAAAGCTGGGTCTTAGAAGAGGTCAATGTCCAAGTCC
Phot2CheckFP	CCATTCCTTCCTACTCTCTACG
Phot2CheckRP	CAATCAATAGCACTTGTATGACCAG
RABE1dFPg	GGGGACAAGTTTGTACAAAAAAGCAGGCTCCATGGCGGTTGCGCCGG
RABE1dRPg	GGGGACCACTTTGTACAAGAAAGCTGGGTCCTAAACGTAACTACAGCAAGCTG
SYP21FPg	GGTGGAGGTATGAGTTTCCAAGATCTCGAAGC
SYP21RPg	GGGGACCACTTTGTACAAGAAAGCTGGGTCTTAGACCAAGACAACGATGATGAC
SYP61FPg	GGTGGAGGTATGTCTTCAGCTCAAGATCCATTC
SYP61RPg	GGGGACCACTTTGTACAAGAAAGCTGGGTCTTAGGTCAAGAAGACAAGAACGAATAG
SITE DIRECTED MUTAGENESIS (PRIMERS)	
Phot2D720NFP	GGACACATAGTATTGGCTAACTTTG
Phot2D720NRP	CAAAGTTAGCCAATACTATGTGTCC

Movie S1: Blue-light-induced phot2 translocation and movement of phot2-labeled punctuate structures in *N. benthamiana* epidermal cell. Two z-scans per minute were performed for a total of 15 min. Images were scanned at a thickness of 1 μm. (Attached avi file)
